# Preparation, characterization and antioxidative activity analysis of recombinant humanized collagen type III-loaded liposomes

**DOI:** 10.1038/s41598-025-07879-6

**Published:** 2025-07-24

**Authors:** Mengxing Du, Chuanxiu Chen, Yuxin Zhou, Yixuan Song, Shan Wang, Shanshan Tang, Jia Yang, Xia Yang, Enli Liu, Yufeng Yu

**Affiliations:** 1 School of Basic Medical Sciences and Pharmacy, Shanxi Medical University, Taiyuan, 030001 China; 2 Shanxi Key Laboratory of Functional Proteins, Shanxi Jinbo Bio-Pharmaceutical Co., Ltd, Taiyuan, 030032 China; 3 Medicinal Basic Research Innovation Center of Chronic Kidney Disease, Ministry of Education, Shanxi Medical University, Taiyuan, 030001 China

**Keywords:** Recombinant type III humanized collagen, Liposomes, Physical stability, Antioxidative activity, Biotechnology, Medical research

## Abstract

To improve the bioavailability and biological activity of recombinant type III humanized collagen (rhCol III), we optimized the preparation process of recombinant type III humanized collagen-loaded liposomes (rhCol III-LIPS) using the film dispersion and periplasmic extrusion methods, with the encapsulation rate as the response value. Then we analyzed the antioxidative activity of rhCol III-LIPS by determining the scavenging rate of free radicals. We investigated the protective effect of rhCol III-LIPS against H_2_O_2_-induced injuries in HaCaT cells by detecting cell vitality, contents of reactive oxygen species (ROS), malondialdehyde (MDA), and activities of antioxidases superoxide dismutase (SOD), catalase (CAT), glutathione peroxidase dismutase (GSH-Px). The results showed, in condition of rhCol III-to-lipid ratio of 1:50, membrane-to-material ratio of 4:1, and ultrasound duration of 20 min, the encapsulation rate of the liposome reached 91.89%±0.59%. The free-radical scavenging rates for 1,1-diphenyl-2-picrohydrazine (DPPH), 2,2-l diazo-bisdiamine salt (ABTS), hydroxyl (OH^−^), and superoxide (O^2−^) anions are 87.11% ± 2.54%, 93.72% ± 2.87%, 86.45% ± 1.62%, 76.67% ± 1.56% respectively. The protective effect on oxidation-damaged cells of rhCol III-LIPS was higher than rhCol III or blank liposomes (B-LIPS). In summary, rhCol III-LIPS has good physical stability and in-vitro antioxidative activity, effectively alleviating H_2_O_2_-induced oxidative injuries in HaCaT cells. Our study provides a more stable and efficient strategy for the application of rhCol.

**Supplementary Information:**

The online version contains supplementary material available at 10.1038/s41598-025-07879-6.

## Introduction

Collagen predominantly exists within skin, bone, teeth, and other tissues of the human body. It represents the most copious and widely dispersed protein in the human body, with its content capable of constituting 30% of the total protein^1–3^. Additionally, it serves as a crucial biomedical material^4–6^, fulfilling the functions of connection, support, repair, and regeneration in human tissues.

At present, collagen is principally classified into two categories: natural collagen and recombinant collagen. Natural collagen is primarily sourced from animal tissues, which harbors a certain degree of risk regarding allergic reactions and diseases and is subject to legal regulations such as those of the European Union. Consequently, the application of biomedical materials based on natural collagen is restricted^7^. Recombinant collagen is procured through the utilization of genetic engineering technology to express the collagen gene^8–12^. The national medical products administration of China has established the guiding principles for naming recombinant collagen biomaterials, which categorizes recombinant collagen materials into three types: recombinant human collagen, recombinant humanized collagen, and recombinant collagen-like protein^13^. Given that the amino acid sequence of recombinant humanized collagen aligns with that of human collagen, in comparison to animal collagen, recombinant humanized collagen exhibits the advantages of low immunogenicity, the absence of viral infection potential, and excellent water solubility^14,15^. In recent years, the technology has experienced rapid advancement and has been successfully implemented in the industries of cosmetics and biomedicine. There has been an increasing number of explorations in other research fields, thereby demonstrating extensive application prospects.

Recombinant humanized collagen is characterized by the full-length or a fragment of amino acid sequence, or the combination of human collagen functional fragments, that encoded by the specific type of human collagen gene. It is capable of being free of any non-human amino acid sequences and any animal-related components through pre-design. As a raw material for implantable medical devices, it can avoid immunogenicity and has the advantages of good biocompatibility, good water solubility and stability. By customizing its design, it is expected to meet precise and personalized medical needs.

Type III collagen, as an infant collagen, has better tissue repair properties compared to type I collagen^16^. Meanwhile, type III collagen is a homologous trimer collagen. Recombinant expression can achieve a structure and function consistent with that of the human body. However, type I collagen is mainly a heterotrimer, and there are still considerable difficulties in reconstituting and expressing type I collagen consistent with that in the human body. Shanxi Jinbo Bio-Pharmaceutical Co., Ltd., as a leading enterprise in recombinant humanized collagen, has completed the large-scale preparation of rhCol III.

rhCol III has been approved for marketing as a Class III medical device in China, and is used for facial filling to correct dynamic wrinkles. In this research direction, two Class III medical device registration certificates have been approved, namely recombinant Type III humanized collagen freeze-dried fiber (National Medical Device Registration No. 20213130488) and recombinant Type III humanized collagen solution for injection (National Medical Device Registration No. 20233131245). It is widely applied in fields such as gynecology, dermatology, and proctology. Therefore, this project selects rhCol III for research.

Free radicals refer to groups or atoms possessing unpaired electrons, which are generated during the metabolic processes of the body as well as upon exposure to the exogenous environment. They exhibit high chemical reactivity. The excessive generation of free radicals within organisms can induce oxidative stress, thereby leading to the impairment of cell or tissue functions and subsequently resulting in various diseases such as inflammation, cancer, and neurodegenerative disorders^17^. Consequently, the effective regulation of oxidative stress serves as a crucial factor in disease prevention. The utilization of antioxidants can effectively modulate the production of free radicals within the body, maintain the homeostatic balance, and attain the objective of preventing and treating diseases^18^. Previous studies have demonstrated that recombinant collagen possesses favorable antioxidant activity. Specifically, its scavenging capacities for DPPH and ABTS free radicals reach 58.91% and 41.21% respectively^19^, thus indicating that recombinant collagen holds the potential to serve as a natural antioxidant. However, in the case of recombinant collagen molecules with high molecular weights, issues such as low transdermal efficiency and low bioavailability arise, and it is of utmost urgency to seek solutions.

Liposomes are closed vesicle structures with an aqueous core composed of phospholipid bilayers. Its phospholipid molecules spontaneously form a bilayer film in aqueous solution, which can encapsulate both hydrophilic and hydrophobic substances. It is often used in fields such as drug delivery and gene transfection, and has good biocompatibility and targeting properties^17,20^. They are capable of forming spherical vesicles possessing a bilayer structure of phospholipid molecules. Liposomes find extensive application in drug formulation. The incorporation of fat-soluble or water-soluble drugs within them can enhance drug solubility and stability, augment permeability and absorption, and fulfill a slow-release function^21,22^. Simultaneously, they can mitigate the adverse reactions induced by drugs and extend the circulation time of drugs within the body, thereby facilitating more efficient drug delivery to the target site^23^. Nevertheless, there is a dearth of pertinent research regarding the capacity of liposomes to encapsulate recombinant humanized collagen.

To improve the bioavailability of rhCol III and give full play to its antioxidant activity, this study prepared it as rhCol III-LIPS. The preparation process was optimized with encapsulation efficiency as the evaluation index, and the quality was evaluated with average vesicles diameter, vesicles size distribution, and electric potential value. The storage stability was investigated and its antioxidative activity in vitro was determined, providing reference for further research and development of collagen products.

## Results and discussion

### Single factor investigation

In order to investigate the effects of rhCol III - lipid ratio, membrane material ratio and ultrasonic time on the encapsulation efficiency of liposomes, we adopted a single-factor experiment to prepare liposomes and determine the encapsulation efficiency.

**Fig. 1:**
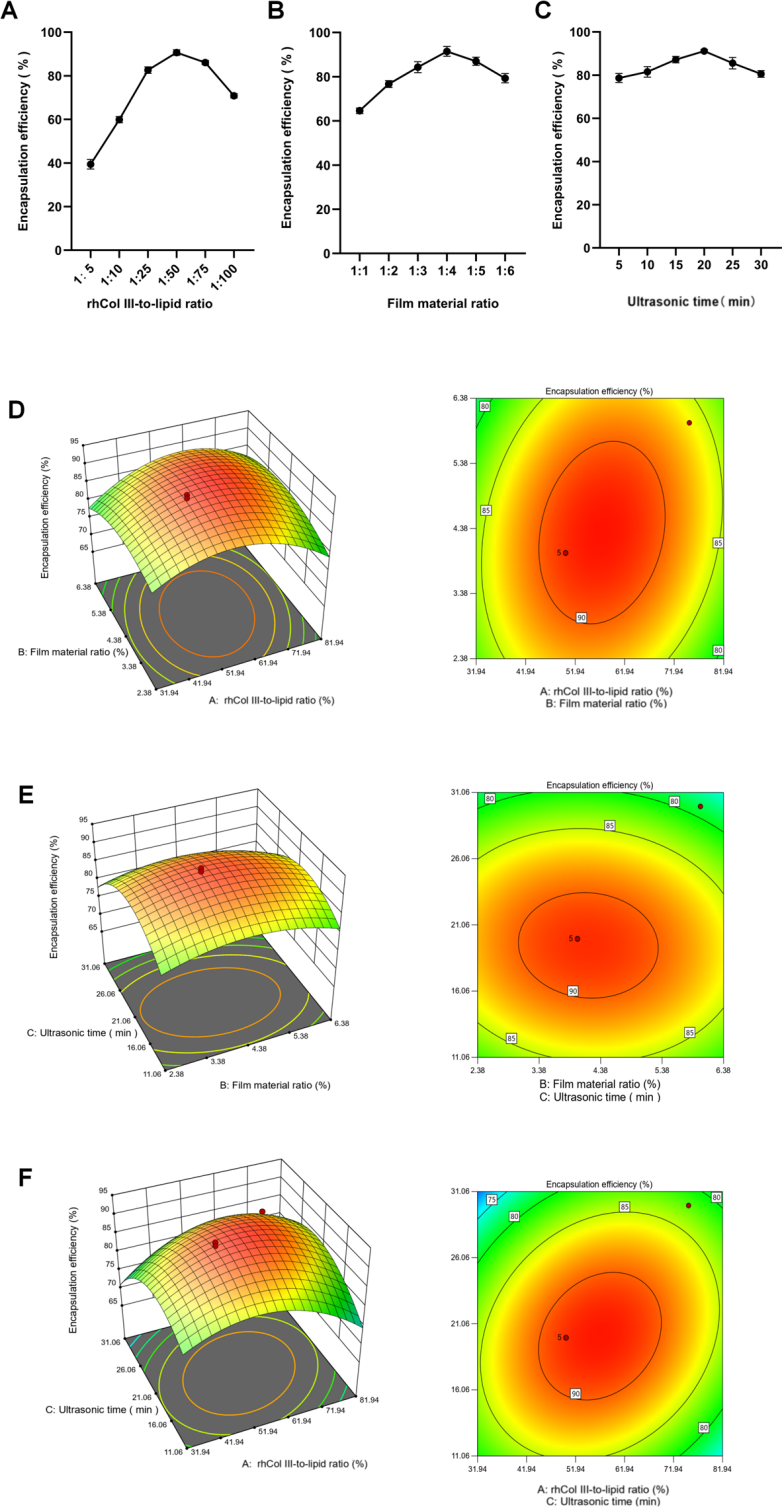
Response surface images of the effect of single factor on liposome encapsulation efficiency and the effect of interaction between two factors on encapsulation efficiency. (**A**) Effect of rhCol III-to-lipid ratio ratio on liposome encapsulation efficiency. (**B**) Effect of membrane-to-material ratio on liposome encapsulation efficiency. (**C**) Effect of ultrasound time on liposome encapsulation efficiency. (**D–F**) Contour lines and response curves of rhCol III-to-lipid ratio vs. membrane-to-material ratio, membrane-to-material ratio vs. ultrasound time, and rhCol III-to-lipid ratio vs. ultrasound time. All statistically relevant data were expressed as mean ± standard deviation, and each experiment was repeated three times in parallel, *N* = 3.

Figure 1A–C shows the effects of different influencing factors on the encapsulation efficiency of rhCol III-LIPS.

With the increase in rhCol III dosage, membrane-to-material ratio, and ultrasound time, the encapsulation efficiency exhibited a trend of first increasing and then decreasing. When the rhCol III-to-lipid ratio was 1:50, the maximum encapsulation efficiency was 90.41%. Therefore, a rhCol III-to-lipid ratio of 1:50 is more suitable (Fig. 1A). When the membrane-to-material ratio was 1:4, the maximum encapsulation efficiency was 92.36%. When cholesterol was added during preparation^24^, the encapsulation efficiency of liposomes gradually increased with the increase of membrane-to-material ratio. After the mass ratio exceeded 1:4, the encapsulation efficiency decreased accordingly. Therefore, a membrane-to-material ratio of 1:4 is more suitable (Fig. 1B). When the ultrasound time was 20 min, the maximum encapsulation efficiency of liposomes was 90.46%; as the ultrasound time prolonged, the encapsulation efficiency of liposomes decreased. Therefore, an ultrasound time of 20 min is more suitable (Fig. 1C).

### Response surface analysis test

To further optimize the preparation conditions of rhCol III-LIPS, three significant influencing factors, namely rhCol III-to-lipid ratio, membrane-to-material ratio, and ultrasound time, were selected to conduct a three-factor three-level response surface analysis test with encapsulation efficiency as the response value. The results are shown in Table 1.

**Table 1:** Test plan and results of response surface analysis.

Test no.	RhCol III-to-lipid ratio A	Membrane-to-material ratio B	Ultrasound time C/min	Encapsulation efficiency Y/%
1	50	2	30	76.44 ± 2.18
2	25	4	30	68.32 ± 1.94
3	25	2	20	77.96 ± 1.67
4	50	4	20	92.31 ± 0.95
5	50	2	10	78.65 ± 1.24
6	50	4	20	91.36 ± 1.18
7	50	6	10	83.12 ± 2.16
8	25	6	20	73.48 ± 1.35
9	50	4	20	90.57 ± 2.42
10	75	2	20	82.16 ± 1.76
11	50	6	30	78.49 ± 1.93
12	75	6	20	85.47 ± 3.42
13	75	4	30	83.42 ± 1.64
14	25	4	10	74.64 ± 1.75
15	50	4	20	92.13 ± 1.59
16	50	4	20	89.79 ± 2.46
17	75	4	10	76.21 ± 1.65

Perform multiple regression fitting on the data in Table 1 using Design-Expert 8.0.6 software to obtain the quadratic regression equation Y = 91.23 + 4.11 A + 0.67B − 0.74 C + 1.95AB + 3.38AC − 0.61BC − 7.50 A^2^ − 3.97B^2^ − 8.09 C^2^ (R^2^ = 0.9775) of rhCol III-LIPS encapsulation efficiency with respect to rhCol III-to-lipid ratio A, membrane-to-material ratio B, and ultrasound time C. The significance analysis test results of the regression equation coefficients are shown in Table 2.

**Table 2:** Variance of regression model and its impact on encapsulation rate.

Source of differences	Sum of squares	Degrees of freedom	Mean square	F value	*P* value	Significance
Model	844.91	9	93.88	33.85	< 0.0001	***
A	135.05	1	135.05	48.70	0.0002	***
B	3.58	1	3.58	1.29	0.2934	
C	4.41	1	4.41	1.59	0.2477	
AB	15.17	1	15.17	5.47	0.0519	
AC	45.70	1	45.70	16.48	0.0048	**
BC	1.46	1	1.46	0.53	0.4911	
A2	236.67	1	236.67	85.34	< 0.0001	***
B2	66.27	1	66.27	23.90	0.0018	**
C2	275.55	1	275.55	99.36	< 0.0001	***
Residual error	19.41	7	2.77			
Lack of fit	14.91	3	4.97	4.42	0.0927	
Pure error	4.50	4	1.13			
Sum	864.33	16				
R^2^					0.9775	
R^2^_adj_					0.9487	
R^2^_Pred_					0.7158	

*P < 0.05, **P < 0.01, ***P < 0.001.

The regression equation depicts the relationship between the encapsulation efficiency of rhCol III-LIPS and diverse influencing factors. According to the significance analysis of the regression model presented in Table 2, the first-order term A, interaction term AC, quadratic terms A^2^ and C^2^ have a highly significant impact on the encapsulation efficiency of rhCol III-LIPS. The quadratic term B^2^ has a significant impact on the encapsulation efficiency of rhCol III-LIPS, whereas the impacts of other factors are insignificant.

The magnitude of the F-value can be utilized to analyze the extent to which the encapsulation efficiency of rhCol III-LIPS is influenced by a single factor. The F-values in the table follow the order: F_A_ > F_C_ > F_B_. The factors influencing the encapsulation efficiency are ranked in descending order as follows: rhCol III-to-lipid ratio, ultrasound time, and membrane-to-material ratio.

Additionally, the significance analysis of the model reveals that the F-value of the corresponding overall model is 33.85 with *P* < 0.0001. The response surface regression model has attained a highly significant level (*P* < 0.01). The lack-of-fit is insignificant (*P* = 0.0927 > 0.05), and the coefficient of variation is 2.03% (< 10%), indicating that non-test factors have a minor effect on the results and the model has good stability.

The correlation coefficient R^2^ of the overall model is 97.75%. The test model fits the actual test well, and approximately 97.75% of the results in the actual test can be accounted for by this fitting model.

The corrected coefficient of determination, R^2^_adj_, is 0.9487, indicating that this model has adequate accuracy and universality, high credibility, and small test errors. Hence, it can be employed to analyze and predict the encapsulation efficiency of rhCol III-LIPS.

### Response surface analysis interaction

The effects of rhCol III-to-lipid ratio, membrane-to-material ratio and ultrasonic time of rhCol III-LIPS on the encapsulation efficiency of rhCol III-LIPS were analyzed using Design Expert 8.0.6 software to obtain the regression equation, and the response surface curves and contour lines of each experimental factor were plotted.

The interaction between rhCol III-to-lipid ratio and ultrasound time had the most significant impact on the encapsulation efficiency of liposomes. In contrast, the interactions between rhCol III-to-lipid ratio and membrane-to-material ratio and between membrane-material ratio and ultrasound time had no significant effects on the encapsulation efficiency of liposomes. When the rhCol III-to-lipid ratio was constant, as ultrasound time prolonged, the encapsulation efficiency of liposomes increased initially and then decreased. When ultrasound time was constant, with the increase in rhCol III-to-lipid ratio, the encapsulation efficiency of liposomes also increased initially and then decreased. The response surface graphic has a steep curve with elliptical and dense contour lines, indicating a highly significant interaction between ultrasound time and rhCol III-to-lipid ratio (Fig. 1D–F).

### Validation of process optimization

The optimal preparation conditions for rhCol III-LIPS were obtained using Design Expert 8.0.6 software with encapsulation efficiency as an indicator, which are as follows: a rhCol III-to-lipid ratio of 1:52.41, a membrane-material ratio of 4.31:1, an ultrasound time of 20.10 min, and an encapsulation efficiency of 91.89%. Based on practical considerations, the optimal preparation conditions were adjusted to a rhCol III-to-lipid ratio of 1:50, a membrane-material ratio of 4:1, and an ultrasound time of 20 min. Under these conditions, rhCol III-LIPS was prepared to verify whether the encapsulation efficiency was at its maximum value. Five repeated tests were conducted, and the results are shown in Table 3.

**Table 3:** Validation results of optimal prescription process.

Number of tests	Vesicles diameter/nm	PDI	Potential/mV	Encapsulation efficiency/%
1	195.73	0.184	−12.23	91.26
2	188.24	0.176	−15.36	92.12
3	191.07	0.198	−13.52	91.31
4	196.10	0.143	−20.12	91.76
5	202.23	0.154	−18.64	92.48
Mean ± SD	194.67 ± 2.91	0.17 ± 0.02	−15.97 ± 3.34	91.79 ± 0.52

The results showed that the rhCol III-LIPS prepared under optimal conditions was close to the predicted maximum encapsulation efficiency of liposomes. It had an average encapsulation efficiency of 91.79%, which was not significantly different from the theoretical prediction value, and a relative standard deviation of 0.52%. This indicates that the response surface methodology-optimized rhCol III-LIPS process is reliable and has certain practical application value.

### Dimensional characterization of rhcol III-LIPS

**Fig. 2:**
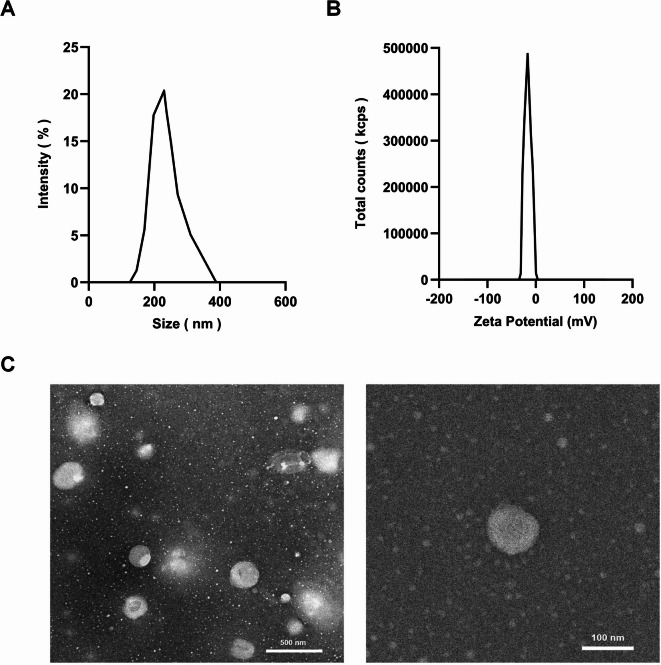
Physicochemical properties and characterization of recombinant humanized collagen type III liposomes. (**A**) Vesicles diameter distribution of recombinant humanized collagen type III liposomes. (**B**) Zeta potential of recombinant humanized collagen type III liposomes. (**C**) Transmission electron microscope (TEM) image of recombinant humanized collagen type III liposomes.

The vesicles size distribution of rhCol III-LIPS is relatively uniform, with an average vesicles diameter of 162.2 ± 7.77 nm and a potential of -31.15 ± 1.48 mV (as shown in Fig. 2A,B). The polydispersion index (PDI) is 0.1844 ± 0.0058. The observation results of transmission electron microscopy showed that rhCol III-LIPS was dispersed, presenting as a single spherical vesicle without obvious aggregation phenomenon. The arrangement was relatively disordered, and the diameter of the vesicle was approximately 160 nm (Fig. 2C).

### Stability investigation

To investigate the storage stability of the freshly prepared rhCol III-LIPS solution, seal it and stored it in the dark at 4 °C. Samples should be taken on the 1st, 3rd, 5th, 7th, 14th, 21st, 28th, 60th, 90th, and 120th days of storage, and their pH value, Zeta potential, and vesicles diameter should be measured.

**Table 4:** Results of stability investigation of rhcol III-LIPS.

Time/day	Appearance and description	pH	Zeta potential/mV	Vesicles Diameter/nm	Visible particles
1	Clear and transparent, milky white in color	6.458 ± 0.24	− 31.15 ± 0.34	162.2 ± 0.67	Free from visible particles
3	Clear and transparent, milky white in color	6.462 ± 0.16	− 35.02 ± 0.26	187.6 ± 0.84	Free from visible particles
5	Clear and transparent, milky white in color	6.472 ± 0.31	− 42.25 ± 0.35	198.3 ± 0.73	Free from visible particles
7	Clear and transparent, milky white in color	6.648 ± 0.14	− 47.15 ± 0.24	204.7 ± 0.88	Free from visible particles
14	Clear and transparent, milky white in color	6.755 ± 0.39	− 42.70 ± 0.42	206.4 ± 0.93	Free from visible particles
21	Clear and transparent, milky white in color	6.516 ± 0.27	− 42.78 ± 0.36	212.3 ± 0.87	Free from visible particles
28	Clear and transparent, milky white in color	6.859 ± 0.15	− 43.34 ± 0.51	216.2 ± 0.67	Free from visible particles
60	Clear and transparent, milky white in color	6.476 ± 0.42	− 46.44 ± 0.37	218.6 ± 0.78	Free from visible particles
90	Clear and transparent, milky white in color	6.383 ± 0.19	− 45.77 ± 0.32	218.7 ± 0.97	Free from visible particles
120	Clear and transparent, milky white in color	6.324 ± 0.23	− 40.18 ± 0.18	205.03 ± 0.95	Free from visible particles

The results showed that the appearance color remained basically unchanged, clear, and transparent. It had a milky white color with a blue milky luster, good fluidity, and no visible foreign matters. The pH value fluctuated within a certain range over time. The absolute value of Zeta potential is greater than 30 mV, indicating that the liposome has good stability. The vesicles diameter increases with time, and the change in vesicles diameter is relatively small after 14 days, as Table 4.

### Analysis of antioxidative activity

**Fig. 3:**
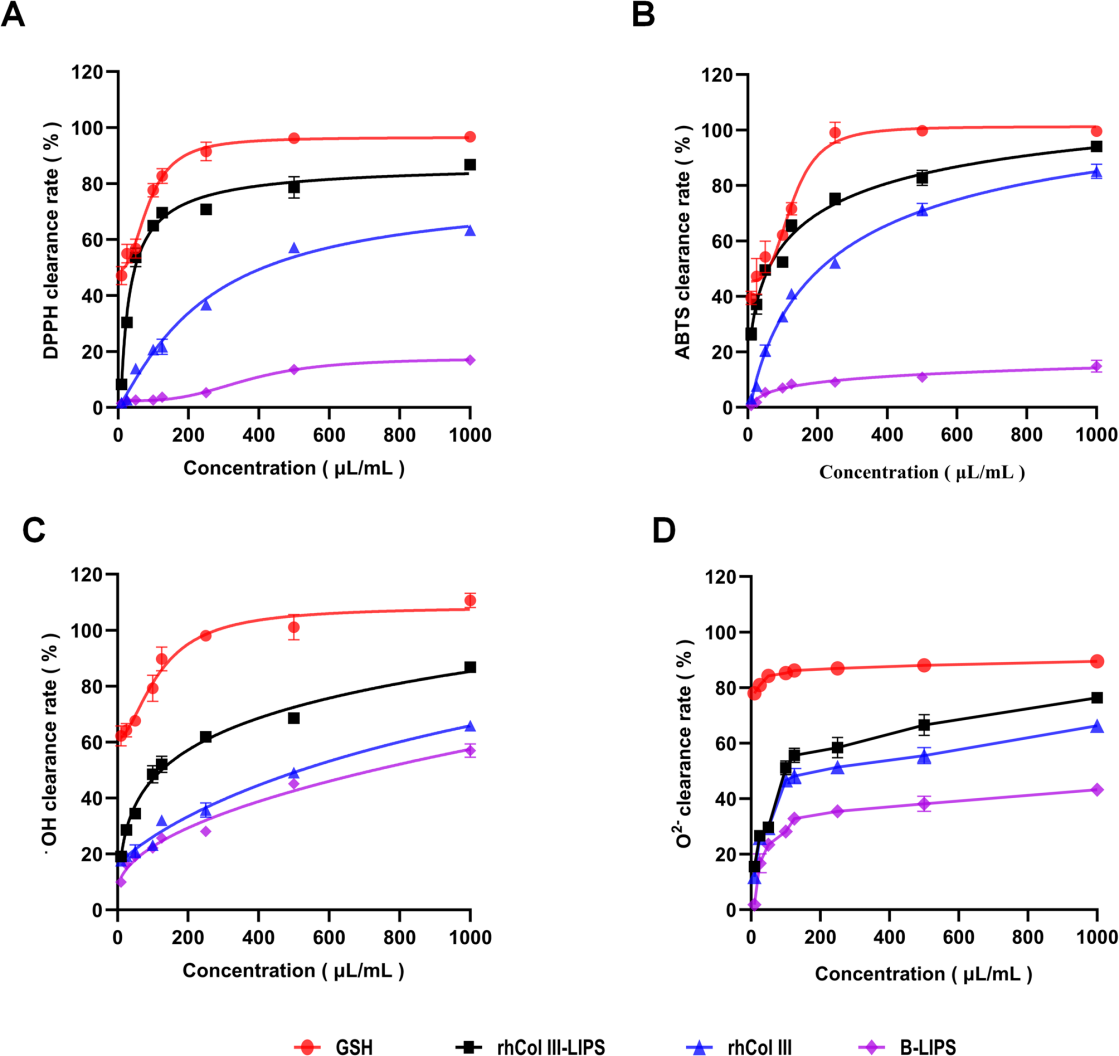
Clearance rates of free radicals by sample solutions with different volume fractions. (**A**) Clearance rate of DPPH radicals. (**B**) Clearance rate of ABTS radicals. (**C**) Clearance rate of hydroxyl radicals. (**D**) Clearance rate of superoxide anion radicals. GSH, reduced glutathione (positive control). rhCol III-LIPS, recombinant humanized collagen type III liposome solution. rhCol III, recombinant humanized collagen type III solution. B-LIPS, blank liposome solution. All statistically relevant data were expressed as mean ± standard deviation, each experiment was repeated three times in parallel, *N* = 3.

To evaluate the antioxidative activity of rhCol III-LIPS, the clearance rates of DPPH, ABTS, hydroxyl, and superoxide anion radicals were measured. The results showed that the clearance abilities of rhCol III-LIPS to DPPH, ABTS, hydroxyl, and superoxide anion radicals had a certain concentration dependence, respectively (Fig. 3A–D), indicating that rhCol III-LIPS is a potential antioxidative component, providing a reference for further research and development of rhCol III products.

### Establishment of H_2_O_2_ induced HaCaT cell oxidative injury model

**Fig. 4:**
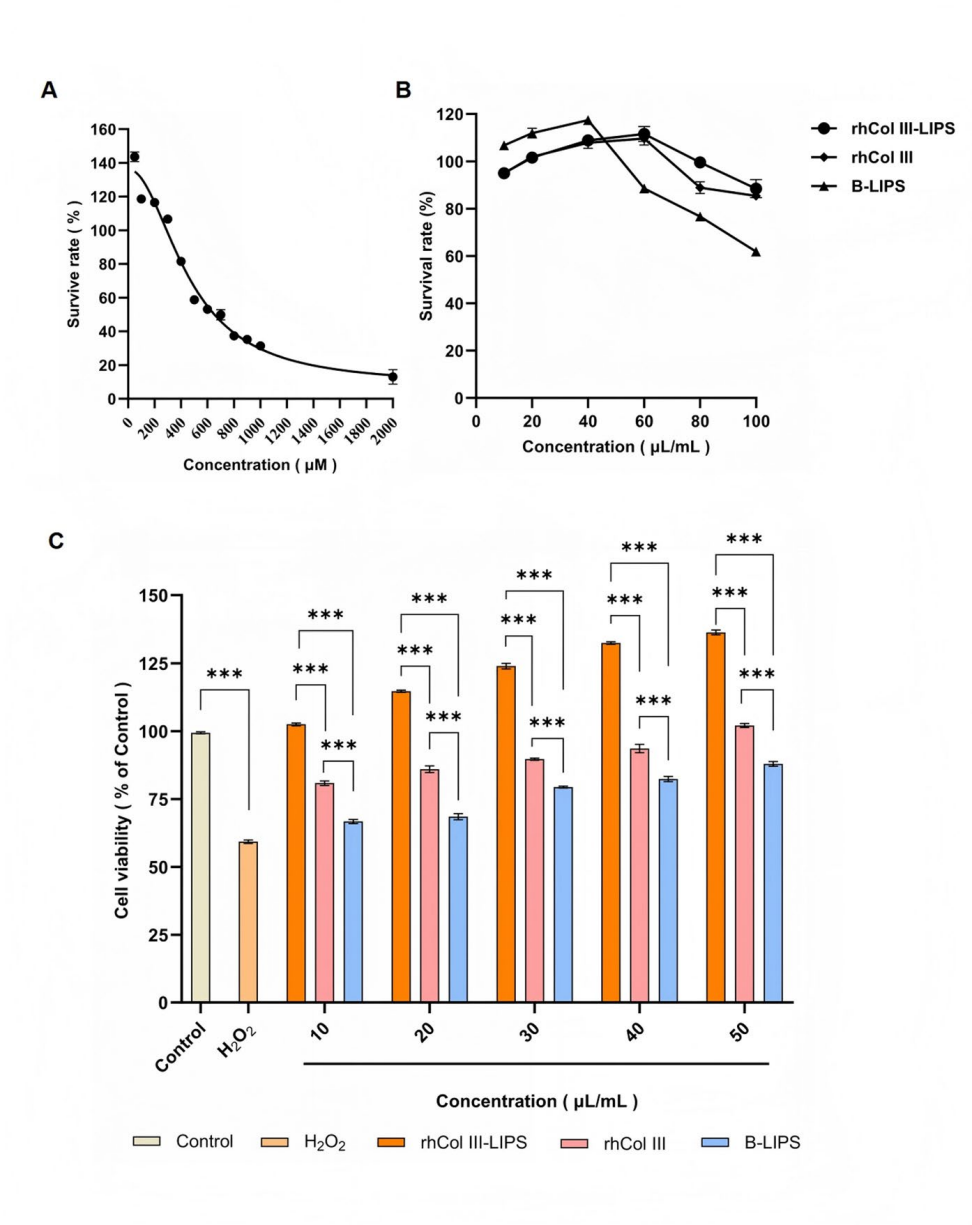
Construction of HaCaT cell oxidative injury model and effects of sample solutions with different volume fractions on HaCaT cells. (**A**) Effect of H_2_O_2_ with different concentrations on the viability of HaCaT cells. (**B**) Effects of rhCol III-LIPS, rhCol III, and B-LIPS with different volume fractions on the viability of HaCaT cells. (**C**) Effects of rhCol III-LIPS, rhCol III, and B-LIPS with different volume fractions on the viability of oxidatively damaged HaCaT cells. All statistically relevant data were expressed as mean ± standard deviation, each experiment was repeated three times in parallel, *N* = 3. Two-way ANOVA was used for statistical comparison between different groups, post-hoc test was used for comparison between groups larger than two, and T-test was used for comparison between two groups. *P < 0.05, **P < 0.01, ***P < 0.001.

The IC_50_ values of H_2_O_2_ on HaCaT cell viability are within the concentration range of 412.1–508.4 µM, respectively (Fig. 4A). When the concentration of H_2_O_2_ was 500 µM, the viability of HaCaT cells decreased to 58.79 ± 0.61% of that of the control group, which was within the range of 40–60%. The cells retained relative viability while being subjected to oxidative stress. Therefore, 500 µM H_2_O_2_ was selected for subsequent tests.

### Cytotoxicity detection on HaCaT cells

When the volume fraction of the sample solution (rhCol III-LIPS, rhCol III and B-LIPS) is below 50 µL/mL, the cell viability of HaCaT cells is above 90%, respectively (Fig. 4B). This can be considered non-toxic and in line with the actual application concentration.

### Protective effect on H_2_O_2_ injury-induced HaCaT cells

The cell viability induced by H_2_O_2_ decreased to 58.26% ± 1.53% of that of the control group (Fig. 4C). Under H_2_O_2_-induced injury condition, the rhCol III-LIPS group demonstrated significantly higher cell viability than the rhCol III group, B-LIPS group. When the concentration of rhCol III-LIPS was increased to 50 µL/mL, the cell viability recovered to 136.47% ± 1.82% of that of the control group, which was significantly different from the H_2_O_2_ group (*P* < 0.001). It can be concluded that rhCol III-LIPS has a significant protective effect on H_2_O_2_ injury-induced HaCaT cells when the concentration is above 50 µL/mL. The rhCol III and B-LIPS at the same concentration also have protective effects on HaCaT cells subjected to oxidative stress, with a significant difference compared with the H_2_O_2_ group.

### Effect on ROS content in HaCaT cells

Reactive oxygen species (ROS) radicals maintain a dynamic balance of continuous production and elimination in the human body and are essential for regulating normal physiological activities of the body^25^. The intracellular level of ROS can directly reflect the level of oxidative injury within cells^25^. The intracellular ROS level can be determined by detecting the fluorescence intensity of cells incubated with DCFH-DA^26^. The series of chain reactions occurring between ROS and lipids is termed lipid peroxidation. It can produce various byproducts that can threaten cellular physiological functions and cause cellular injury^27^.

**Fig. 5:**
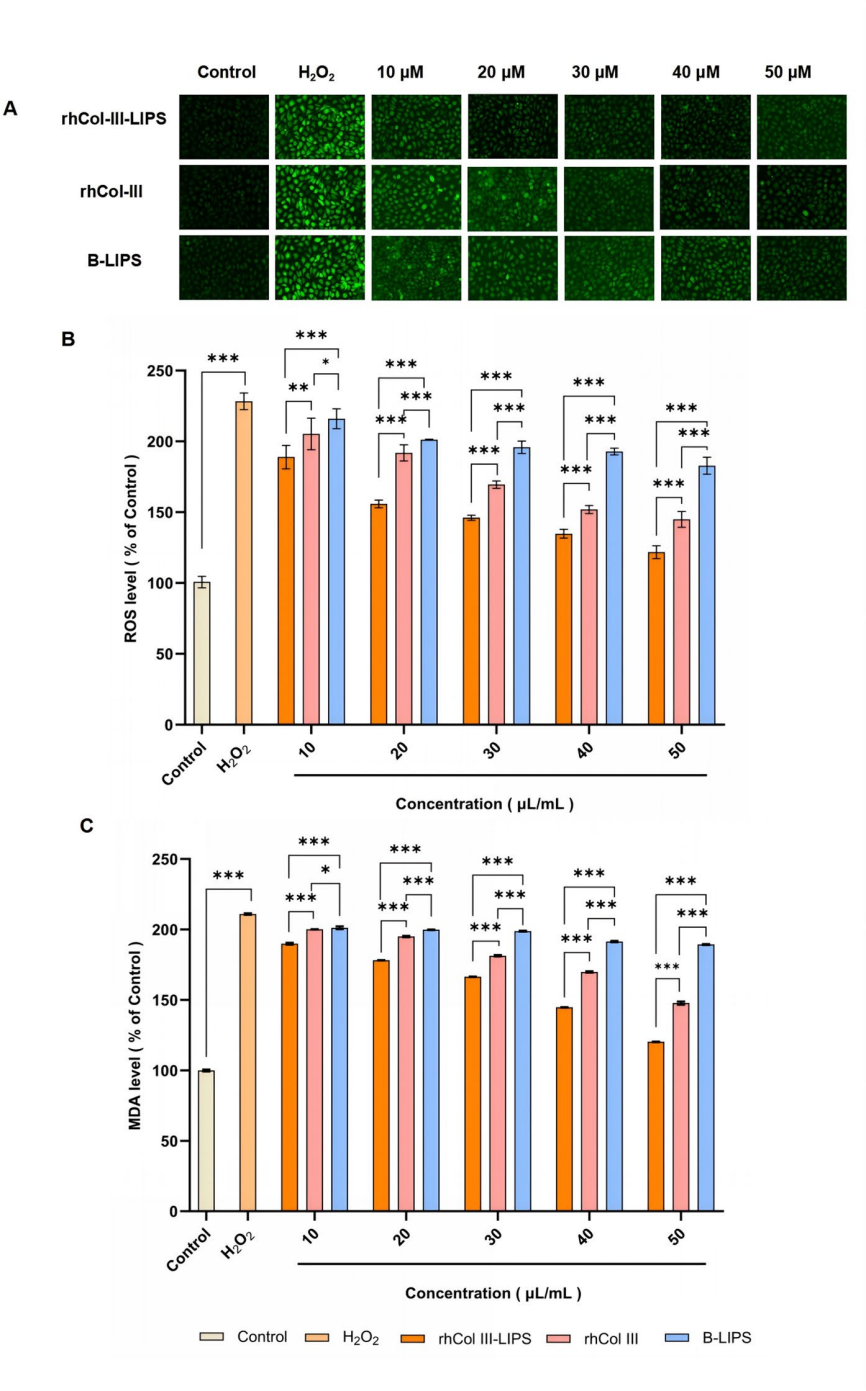
Effects of sample solutions with different volume fractions on ROS and MDA contents in HaCaT cells. (**A**) Fluorescence images of HaCaT cells treated with H_2_O_2_ and different volume fraction sample solutions. (**B**) Effects of rhCol III-LIPS, rhCol III, and B-LIPS with different volume fractions on ROS content in HaCaT cells. ImageJ was used to perform data analysis on (**A**). (**C**) Effects of rhCol III-LIPS, rhCol III, and B-LIPS with different volume fractions on MDA content in HaCaT cells. All statistically relevant data were expressed as mean ± standard deviation, each experiment was repeated three times in parallel, *N* = 3. Two-way ANOVA was used for statistical comparison between different groups, post-hoc test was used for comparison between groups larger than two, and T-test was used for comparison between two groups. *P < 0.05, ***P < 0.001.

The rhCol III-LIPS group demonstrated a significantly stronger capacity to reduce ROS than the rhCol III and B-LIPS groups at all tested concentration. When HaCaT cells were treated with 500 µM H_2_O_2_, the intracellular level of ROS significantly increased (Fig. 5A,B). After being treated with H_2_O_2_, HaCaT cells underwent an oxidative stress response, resulting in an increase in ROS content. After treatment with sample solutions of different volume fractions, the ROS content significantly decreased. The degree of decrease increased with the increase in volume fraction, indicating that the regulation of ROS levels by sample solutions has a dose-dependent effect.

When the volume fraction of rhCol III-LIPS was 50 µL/mL, the fluorescence intensity decreased by 45% compared with the H_2_O_2_ group, and to 1.24 times that of the control group. This was similar to some previous studies^27–29^indicating that the antioxidant injury ability of rhCol III-LIPS is superior to that of rhCol III and B-LIPS.

### Effect on MDA content in HaCaT cells

After treatment with H_2_O_2_ for oxidative injury, the MDA content in HaCaT cells increased to 2.12 times that of the control group (Fig. 5C). This was significantly different from the control group (*P* < 0.001), indicating severe oxidative injury to the cells. rhCol III-LIPS, rhCol III, and B-LIPS all dose-dependently reduced H_2_O_2_-induced MDA levels. rhCol III-LIPS and rhCol III were significantly more effective than B-LIPS at all tested concentrations. Furthermore, rhCol III-LIPS outperformed rhCol III at all administered volume fraction. RhCol III-LIPS in the sample solution had a certain ameliorative effect on the excessive lipid peroxidation caused by oxidative injury in HaCaT cells.

### Effect on the antioxidase activity in HaCaT cells

Common antioxidases such as CAT, SOD, and GSH-Px play important roles in the cellular redox balance mechanism^30,31^. The level of antioxidase activity reflects the severity of oxidative injuries endured by cells. Intracellular antioxidase activity can serve as an indicator of a cell’s ability to tolerate antioxidative injuries.

**Fig. 6:**
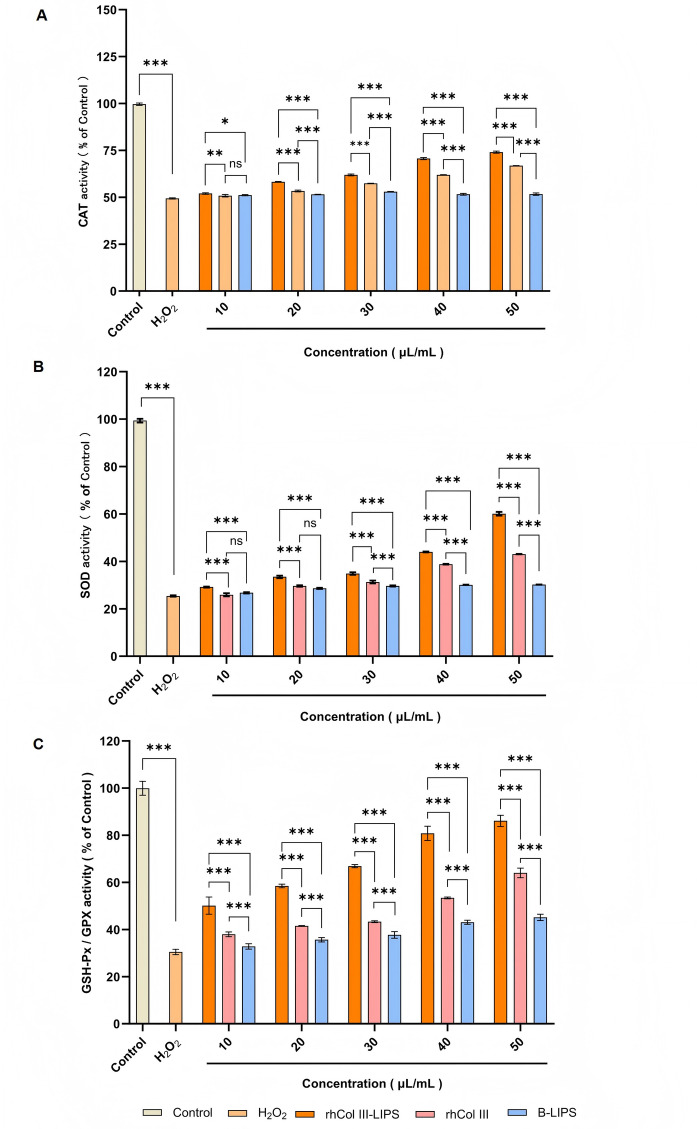
Effect of sample solutions with different volume fractions on antioxidase activity in HaCaT cells. **(A**) Effects of rhCol III-LIPS, rhCol III, and B-LIPS with different volume fractions on CAT activity in HaCaT cells. (**B**) Effects of rhCol III-LIPS, rhCol III, and B-LIPS with different volume fractions on SOD activity in HaCaT cells. (**C**) Effects of rhCol III-LIPS, rhCol III, and B-LIPS with different volume fractions on GSH-Px activity in HaCaT cells. All statistically relevant data were expressed as mean ± standard deviation, each experiment was repeated three times in parallel, *N* = 3. Two-way ANOVA was used for statistical comparison between different groups, post-hoc test was used for comparison between groups larger than two, and T-test was used for comparison between two groups.ns indicates no significant difference, *P < 0.05, **P < 0.01, ***P < 0.001.

Treated with H_2_O_2_, the CAT activity decreased to 40.42% ± 2.31% of that of the control group. After treatment with rhCol III-LIPS, the CAT activity increased significantly (*P* < 0.01) (Fig. 6A). When the volume fraction of rhCol III-LIPS increased to 50 µL/mL, it recovered to 74.23% ± 1.67% of that of the control group. RhCol III and B-LIPS had a certain promoting effect on CAT activity after oxidative injury occurred. At a volume fraction of 50 µL/mL, CAT activity recovered to 66.69% ± 1.72% and 51.60% ± 1.86% of that of the control group, respectively. This indicates that rhCol III-LIPS has a better promotion effect on CAT activity.

Treated with H_2_O_2_, the SOD activity decreased to 25.47% ± 2.51% of that of the control group, indicating strong oxidative injury to the cells induced by H_2_O_2_ (Fig. 6B). After treatment with rhCol III-LIPS, the SOD activity significantly increased (*P* < 0.001). When the volume fraction increased to 50 µL/mL, the SOD activity recovered to 60.24% of that of the control group. RhCol III and B-LIPS also had a promoting effect on SOD activity after oxidative injury occurred. However, the degree was lower than that of rhCol III-LIPS.

HaCaT cells were treated with H_2_O_2_, the enzyme activity of GSH-Px decreased to 31.97% ± 3.15% of that of the control group, indicating a severe decrease in cellular antioxidant levels induced by H_2_O_2_ (Fig. 6C). Treated with rhCol III-LIPS, the GSH-Px enzyme activity increased significantly (*P* < 0.001). When the volume fraction of rhCol III-LIPS increased to 50 µL/mL, it recovered to 86% of that of the control group. Treated with rhCol III and B-LIPS at the volume fraction of 50 µL/mL, the GSH-Px enzyme activity increased to 64.02% ± 2.17% and 45.14%±1.92% of that of the control group. This indicates that rhCol III-LIPS has the strongest promoting effect on GSH-Px enzyme activity at the same volume fraction.

In summary, compared with normal cultured cells, the ROS and MDA content of oxidatively damaged cells increased significantly. After treatment with rhCol III-LIPS, rhCol III, and B-LIPS, the ROS and MDA content of the rhCol III-LIPS group decreased significantly in a concentration-dependent manner compared to the injury group. Compared with normal incubated cells, the activities of antioxidases CAT, SOD, and GSH-Px in oxidatively injured cells were significantly reduced. After treatment with rhCol III-LIPS, rhCol III, and B-LIPS, the antioxidant enzyme activity in the rhCol III-LIPS group increased significantly in a concentration-dependent manner compared to the injury group. At the same volume fraction, the protective effect of rhCol III-LIPS on oxidatively damaged cells was stronger than that of rhCol III and B-LIPS. This indicates that rhCol III-LIPS has higher antioxidative activity.

## Materials and methods

Recombinant type III humanized collagen freeze-dried fiber was provided by Shanxi Jinbo Biopharmaceutical Co., LTD. (Shanxi, China). Hydrogenated lecithin was supplied by Suzhou Nakang Biotechnology Co., LTD. (Suzhou, China). Cholesterol, Tween-80, and potassium persulfate were obtained from Sinopharm Group Chemical Reagent Co., LTD. Anhydrous ethanol was purchased from Tianjin Fengchuan Chemical Reagent Technology Co., LTD. (Tianjin, China). Hydrogen peroxide and ferrous sulfate heptahydrate were from Xilong Science Co., LTD. DPPH, ABTS, nitroblue tetrazolium chloride (NBT), nicotinamide adenine dinucleotide hydrogen (NADH), phenazine methosulfate (PMS), and glutathione were provided by Beijing Solaibao Technology Co., LTD. (Beijing, China). CCK-8 was purchased from Nanjing Nuoweizan Biotechnology Co., LTD. (Nanjing, China). Fetal bovine serum (FBS), Dulbecco’s modified eagle medium (DMEM), penicillin, streptomycin, and trypsin-EDTA were obtained from Gibco (Gibco, Shanghai, China). The BCA assay kit was purchased from Shanghai Beyotime Biotechnology Co., Ltd. The DCFH-DA fluorescent probe was purchased from Beijing Solarbio Science & Technology Co., Ltd. The MDA content assay kit and the SOD, CAT, and GSH-Px viability assay kits were provided by Beijing Solarbio Science & Technology Co., Ltd. (Beijing, China).

### Cell culture

Human immortalized epidermal cells (HaCaT cells, provided by the Shanxi Key Laboratory of Functional Protein) were cultured in DMEM medium containing high sugar (4.5 g/L), 10% fetal bovine serum, 100 U/mL penicillin, and 100 µg/mL streptomycin. The cells were incubated at 37 °C in CO_2_-95% air-humidified incubators. All experiments were conducted during the logarithmic phase of cell growth.

### Preparation of rhCol III-LIPS

RhCol III-LIPS was prepared by the film dispersion method and the membrane extrusion method. Weigh 0.48 g of hydrogenated lecithin and 0.12 g of cholesterol respectively. Dissolve them in absolute ethanol and evaporate under reduced pressure with a 50 °C vacuum rotary evaporator (Shanghai Yarong Biochemical Instrument Factory) to form a uniform lipid membrane. After vacuum drying to constant weight, add 0.008 g of rhCol III lyophilized powder, 0.5 mL of 1% Tween-80, and 100 mL of ultrapure water. Then, hydrate the lipid membrane at room temperature (25 °C). Filter the newly formed liposome suspension through a 0.22 μm aqueous microporous filter membrane and store it at 4 °C under light protection. Blank liposomes (B-LIPS) were prepared by the same method as a control.

### Determination of encapsulation efficiency

The free rhCol III was removed from the rhCol III-LIPS solution by dialysis. The freshly prepared rhCol III-LIPS solution was transferred into a dialysis tube (100 kDa) and dialyzed against PBS buffer solution (pH 7.4) for 24 h to remove the free rhCol III. Demulsification was performed on rhCol III-LIPS before and after dialysis with TritonX-100 solution separately to release the rhCol III embedded in liposomes. The collagen concentration was determined using a BCA assay kit. The rhCol III concentration was determined at a wavelength of 562 nm using a microplate reader (Biotek Synergy H1, USA), which was denoted as Wsum and W respectively. The encapsulation efficiency (%) was calculated using the following formula:

$$\:\text{Encapsulation\:efficiency(\%)=}\frac{{\text{W}}_{\text{sum}}}{\text{W}} \times {100}$$

### Single factor study of Preparation technology

With other conditions kept constant, and using the rhCol III-LIPS encapsulation efficiency as an indicator, factors such as rhCol III-to-lipid ratio, membrane-to-material ratio, and ultrasound time were investigated and screened separately under the following conditions. Three parallel tests were conducted at each level, and the average was calculated.

The mass ratios of rhCol III to hydrogenated lecithin were 1:5, 1:10, 1:25, 1:50, 1:75, and 1:100, respectively. The mass ratios of hydrogenated lecithin to cholesterol were 1:1, 1:2, 1:3, 1:4, 1:5, and 1:6, respectively. Ultrasound time: 5, 10, 15, 20, 25, and 30 min.

### Response surface analysis test

Three significant factors, namely rhCol III-to-lipid ratio, membrane-to-material ratio, and ultrasound time, were selected, and with encapsulation efficiency as the response value, a three-factor three-level response surface analysis test was conducted using Design Expert 8.0.6 software. The test design is presented in Table 5. Based on the results of the response surface analysis, the optimal preparation process of rhCol III-LIPS was predicted and three sets of samples were prepared. The encapsulation efficiency was compared with the predicted values.

**Table 5:** Design factors and levels of response surface analysis.

Factor	Level		
	− 1	0	1
rhCol III-to-lipid ratio A	1:25	1:50	1:75
Membrane-to-material ratio B	1:2	1:3	1:4
Ultrasound time C	1:10	1:15	1:20

### Dimensional characterization of rhcol III-LIPS

The average vesicles size, size distribution, and Zeta potential of liposomes were measured using Malvern Zetasizer Nano ZSU3100 (Mslvern Instruments, Malvern, UK). The size distribution was represented by the polydispersity index (PDI).

### Micromorphological observations

Observe the morphology of liposomes under 120 kV using a transmission electron microscope (TEM, Motic). Drop 10 µL of rhCol III-LIPS solution onto the surface of the carbon-coated support grid to allow for full adsorption. Then, absorb the excess liquid with filter paper to prepare the TEM samples. Take an appropriate amount of phosphotungstic acid staining solution for staining. Then, dry the sample at room temperature.

### Investigation of stability

Store the freshly prepared rhCol III-LIPS solution at 4 °C under light protection. Observe and determine its appearance, color, pH value, vesicles size, Zeta potential, and the presence of visible particles at 1, 3, 5, 7, 14, 21, 28, 60, 90, and 120 days.

### Determination of the clearance ability for DPPH radicals

Take 1.5 mL of DPPH working solution. Add 1 mL of sample solutions with different volume fractions, mix well. Let the mixture react for 30 min under light protection. Then, centrifuge the mixture, transfer the supernatant, and measure the absorbance at 517 nm on a microplate reader.

For the control group, ddH_2_O is used instead of the sample solution. For the blank group, absolute ethanol is used instead of the DPPH working solution^32^.

### Determination of the clearance ability for ABTS radicals

Transfer 200 µL of ABTS working solution into a 96-well plate. Add 10 µL of sample solutions with different volume fractions and mix well. Then, incubate the mixture under light protection at room temperature. Finally, measure the absorbance at 734 nm with a microplate reader.

For the control group, ddH_2_O is used instead of the sample solution. For the blank group, PBS is used instead of the ABTS working solution.

### Determination of the clearance ability for hydroxyl radicals

Take a centrifuge tube. Add 100 µL of salicylic acid solution and 100 µL of FeSO_4_ solution, and mix thoroughly. Then, add 100 µL of sample solutions with different volume fractions and 100 µL of H_2_O_2_ solution. Mix well and incubate at 37 °C. After cooling, measure the absorbance at 536 nm.

For the control group, ddH_2_O is used instead of the sample. For the blank group, ddH_2_O is used instead of the salicylic acid solution^33^.

### Determination of the clearance ability for superoxide anion radicals

Transfer 1 mL of sample solutions with different volume fractions into a centrifuge tube. Then, add 1 mL of NBT solution and 1 mL of NADH solution. Mix well. After that, add 1 mL of PMS solution.

Incubate the mixture at room temperature under light protection. Then, measure the absorbance at 560 nm with a microplate reader. For the control group, ddH_2_O is used instead of the sample^34^.

### Calculation methods

The clearance rate of DPPH, ABTS, and hydroxyl radicals was calculated as follows:

$$\:\text{Clearance\:rate\:of\:radicals (\:\%)=}\frac{\text{Ac+Ab-As}}{\text{Ac}} \times {100}$$

where A_C_ represents the absorbance of the control group, A_B_ represents the absorbance of the blank group, and A_S_ represents the absorbance of the sample group.

The clearance ability for superoxide anion radicals was calculated as follows:

$$\:\text{Clearance\:rate\:of\:superoxide\:anions\:radicals(\:\%)=}\frac{\text{Ac\:-\:As}}{\text{Ac}} \times{100}$$

where A_C_ represents the absorbance of the control group and A_S_ represents the absorbance of the sample.

### Establishment of H_2_O_2_ induced oxidative injuries model in HaCaT cells

HaCaT cells originate from the human epidermis and are closely related to the physiological functions of the skin. As the largest organ of the human body, the skin is directly exposed to the external environment and is prone to oxidative stress. Secondly, by constructing an oxidative damage model for HaCaT cells, the oxidative stress process that occurs when the skin is stimulated by external factors such as ultraviolet rays and pollution can be simulated. Meanwhile, HaCaT cells have immortalized characteristics and can be stably subcultured in vitro, providing a large number of uniform cells for experimental research and ensuring the repeatability of experimental results.

Incubate the HaCaT cells until they reach a confluence degree of 80−90%. Then, inoculate the cells into a 96-well plate at a density of 5 × 10^3^ cells/well. Culture the cells for 24 h to allow them to adhere to the wall and grow. Subsequently, perform starvation culture for 24 h.

Divide the cells into 14 groups. Add serum-free and double antibody-free basic medium containing 50, 100, 200, 300, 400, 500, 600, 700, 800, 900, 1000, and 2000 µM of H_2_O_2_, respectively, to each group and induce injuries for 2 h. The control group is cultured with medium. After 2 h, discard the medium and wash with PBS solution. Then, add the medium containing CCK-8 solution. Add the reagent in the same volume to the blank group.Incubate the cells at 37 °C for 2 h. Then, measure the absorbance at 450 nm. Calculate the cell viability according to the following formula:

$$\:\text{Cell\:viability(\:\%)=}\frac{\text{As\:-\:Ab}}{\text{Ac\:-\:Ab}} \times {100}$$

where, Ac represents the absorbance of the control group, Ab represents the absorbance of the blank group, and As represents the absorbance of the sample group.

### Effect on HaCaT cell viability

Incubate the cells in a 96-well plate. After 24 h, divide the cells into three groups. Add the media containing rhCol III-LIPS, rhCol III, and B-LIPS at volume fractions of 10, 20, 30, 40, and 50 µL/mL, respectively. Incubate the cells for another 24 h. Then, discard the media, wash the cells with PBS, and determine the cell viability.

### Protective effect against H_2_O_2_ injury-induced HaCaT cells

Inoculate cells at a density of 5 × 10^3^ cells/well into 96-well plates. Culture the cells for 24 h and then divide them into three groups: ① control group: normal culture; ② injury group: After 24 h of normal culture, induce cells to undergo oxidative injury using serum-free and double antibody-free basic medium containing 500 µM of H_2_O_2_; ③ sample group: Based on the injury group, add basic medium containing different volume fractions of rhCol III-LIPS, rhCol III, and B-LIPS and incubate for 24 h.

After completing the incubation and stimulation process, discard the medium. Wash the cells with PBS and determine the cell viability.

### Effects of different sample solutions on ROS content in HaCaT cells

Inoculate HaCaT cells at a density of 3 × 10^5^ cells/well into a 6-well plate. Incubate the cells normally for 24 h and then divide them into three groups: control group, injury group, and normal group for testing. Treat the control group and injury group in the same manner as the aforementioned groups. Set five concentration gradients for the sample group (rhCol III-LIPS, rhCol III, and B-LIPS).

After inducing oxidative injury to cells, wash the cells with PBS. Then, add 1 mL of basic medium and 1 µL of DCFH-DA fluorescent probe to each well to achieve a final concentration of 10 µM for the fluorescent probe. After 20 min of incubation, discard the medium. Wash three times with basic medium to thoroughly rinse off the probe. Add 1 mL of PBS. Observe under a fluorescence microscope and calculate the fluorescence intensity using ImageJ software.

### Effects of different sample solutions on the MDA content and antioxidase activity in HaCaT cells

Inoculate HaCaT cells at a density of 3 × 10^5^ cells/well into a 6-well plate. Incubate the cells for 24 h with complete medium and then divide them into three groups: control group, injury group, and normal group for testing. Treat the control group and injury group in the same manner as the above groups. Set five concentration gradients for the sample group (rhCol III-LIPS, rhCol III, and B-LIPS).

After inducing oxidative injury to cells, collect the cells. Add extraction solution and conduct ultrasonication to the cells. Take the supernatant and place it on ice for testing. Then, measure according to the instructions of the MDA content assay kit, and the SOD, CAT, and GSH-Px viability assay kits.

### Statistical method

Statistical analysis was conducted using GraphPad Prism 9.0 software to analyze the significance of differences between experimental data. All data involved in statistics were expressed as mean ± standard deviation, and each set of experiments was repeated three times in parallel. Statistical comparisons between different groups were performed using one-way analysis of variance, a post facto test for groups larger than 2, or a T-test for comparisons between two groups. A P-value of less than 0.05 was considered statistically significant.

## Conclusion

This study employed a thin-film dispersion method combined with membrane extrusion to prepare rhCol III-LIPS. The process parameters were optimized through single-factor experiments and response surface methodology, yielding the following optimal conditions: collagen-to-lipid mass ratio of 1:50, membrane material mass ratio of 4:1, and ultrasonication duration of 20 min. Under these conditions, the rhCol III-LIPS demonstrated an exceptional encapsulation rate of 91.89 ± 0.59%, consistent with previous reports on the high encapsulation rate achieved by thin-film dispersion methods^35^and surpassing traditional liposome preparation techniques^36^ .

Characterization using Malvern nanoparticle analysis and transmission electron microscopy (TEM) revealed that the rhCol III-LIPS exhibited a uniform spherical morphology with a homogeneous particle size distribution. The average hydrodynamic diameter was 162.2 ± 7.77 nm, zeta potential was − 31.15 ± 1.48 mV, and polydispersity index (PDI) was 0.1844 ± 0.0058, indicating excellent colloidal stability and dispersion. The particle size aligns with the optimal range (100–200 nm) for drug delivery systems, while the low PDI further validates the efficacy of membrane extrusion in enhancing size uniformity^37,38^. The negative zeta potential contributes to electrostatic stabilization, minimizing particle aggregation^39^.

Cytotoxicity assays confirmed the biocompatibility of rhCol III-LIPS, consistent with the low toxicity inherent to phospholipid-based delivery systems^40^. In vitro antioxidant studies demonstrated superior radical scavenging capacities for DPPH (87.11 ± 2.54%), ABTS (93.72 ± 2.87%), hydroxyl (86.45 ± 1.62%), and superoxide anion (76.67 ± 1.56%), significantly outperforming free rhCol III and B-LIPS. These results underscore the role of liposomes in protecting collagen from degradation while enhancing its antioxidant activity^41^.

In HaCaT oxidative stress models, rhCol III-LIPS restored cell viability from 59.28 ± 0.65% to 136.36 ± 0.82%, reduced intracellular ROS levels from 222.77 ± 5.89% to 121.7 ± 4.46%, decreased MDA content from 210.86 ± 0.65% to 120.22 ± 0.51%, and upregulated antioxidant enzyme (CAT, SOD, GSH-Px) activities. These effects were markedly superior to those of free rhCol III and B-LIPS, attributable to the liposomes’ targeted delivery and sustained-release properties^42,43^ .

Compared to free rhCol III, rhCol III-LIPS exhibited enhanced stability and antioxidant efficacy, corroborating literature on liposomes’ ability to improve bioactive molecule stability and bioavailability^44^. This study provides critical process parameters and theoretical foundations for rhCol III-based product development, while offering novel insights for antioxidant-functionalized formulations. Future investigations should focus on in vivo mechanisms and long-term stability to facilitate clinical translation and industrial scalability.

## Electronic supplementary material

Below is the link to the electronic supplementary material.

Supplementary Material 1

## Data Availability

All data generated or analysed during this study are included in this published article (and its Supplementary Information files).
