# Supplementary material for: Preparation, characterization and antioxidative activity analysis of recombinant humanized collagen type III-loaded liposomes
Source: Sci Rep. 2025 Jul 24;15:26907. doi: 10.1038/s41598-025-07879-6 (PMC12289889; doi:10.1038/s41598-025-07879-6)
Supplement: Supplementary file 1 — Supplementary Material 1 [file 41598_2025_7879_MOESM1_ESM.pptx]

## Slide 1
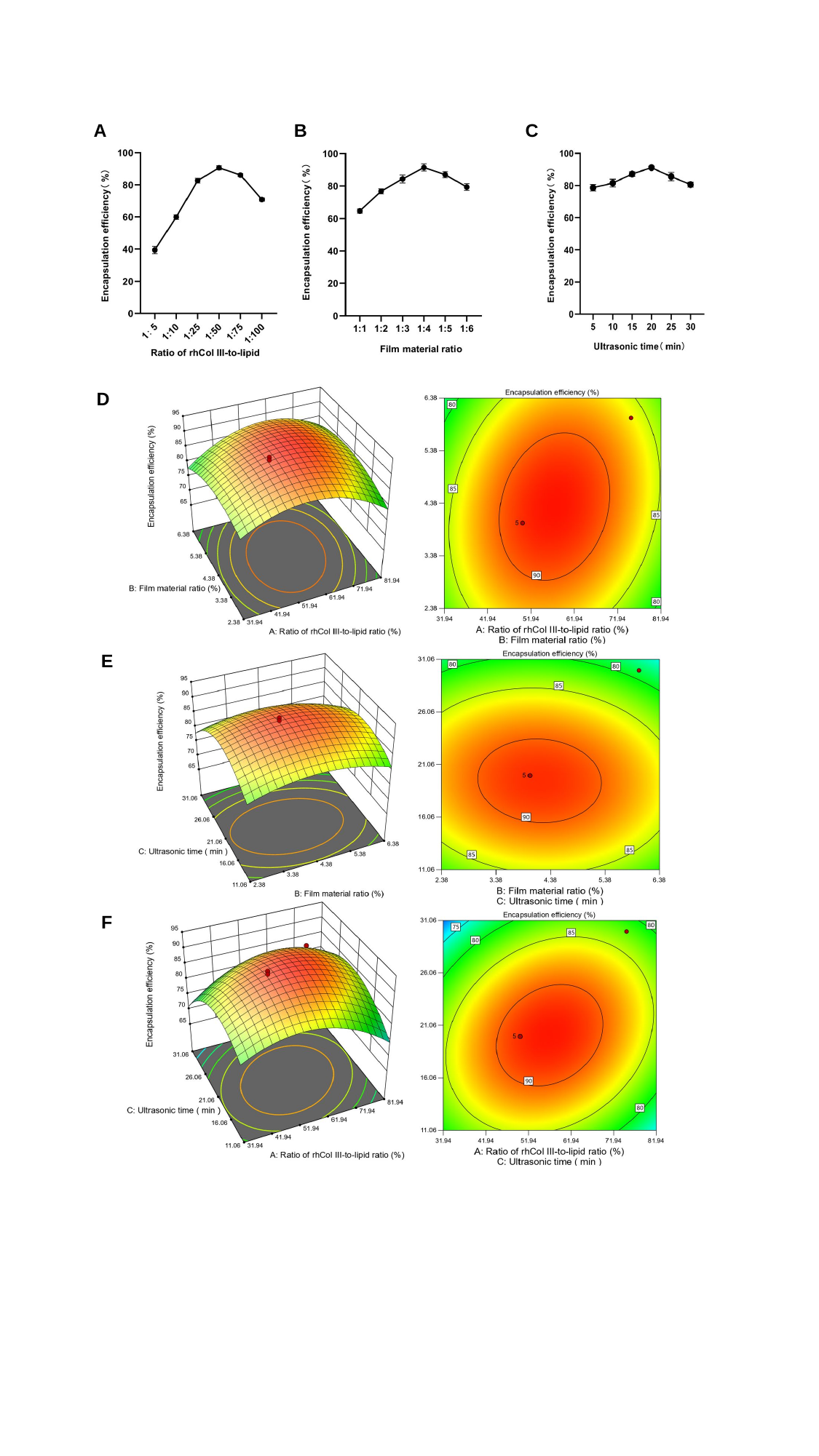

A B C
D
E
F

## Slide 2
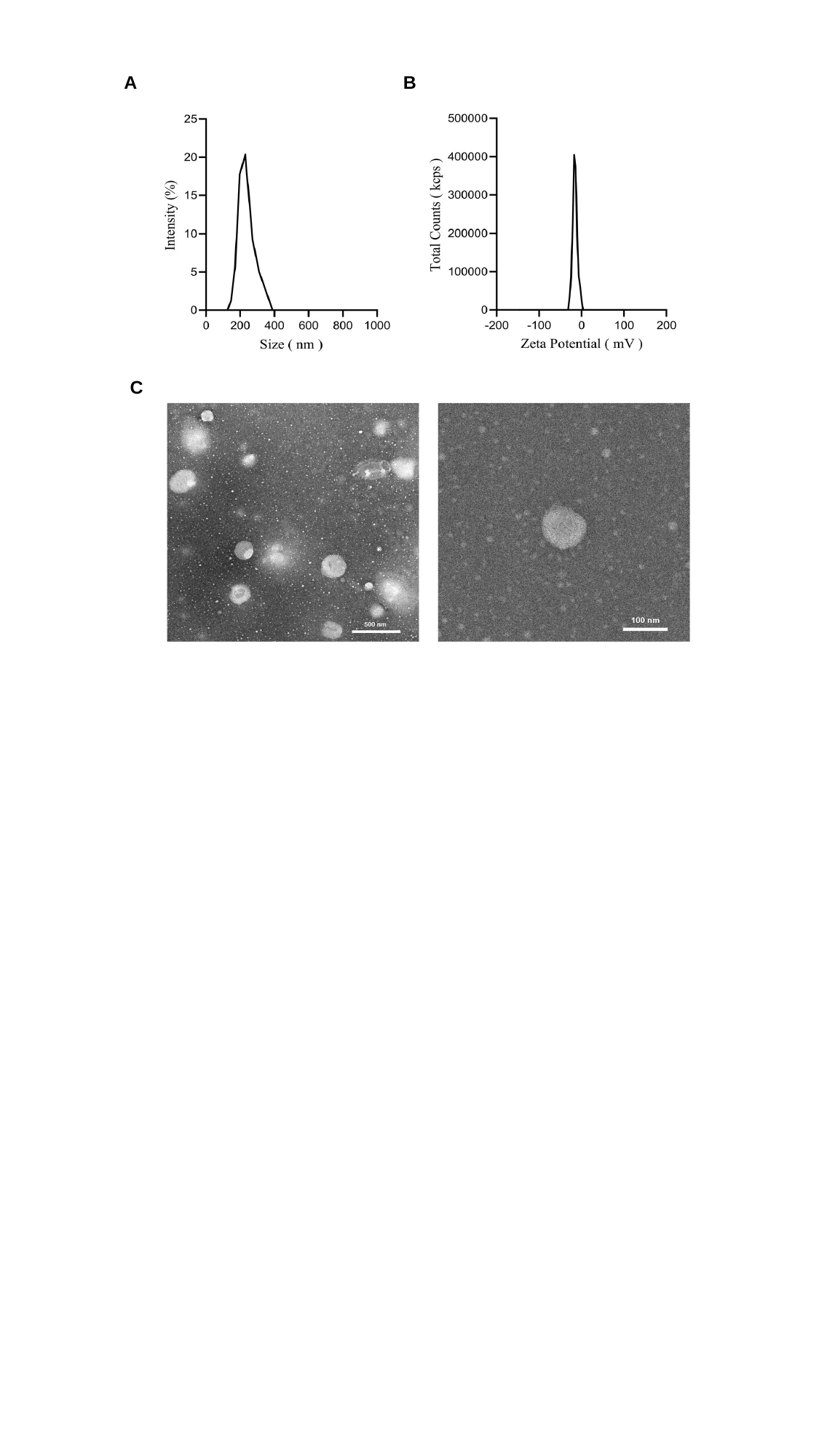

A
B
C

## Slide 3
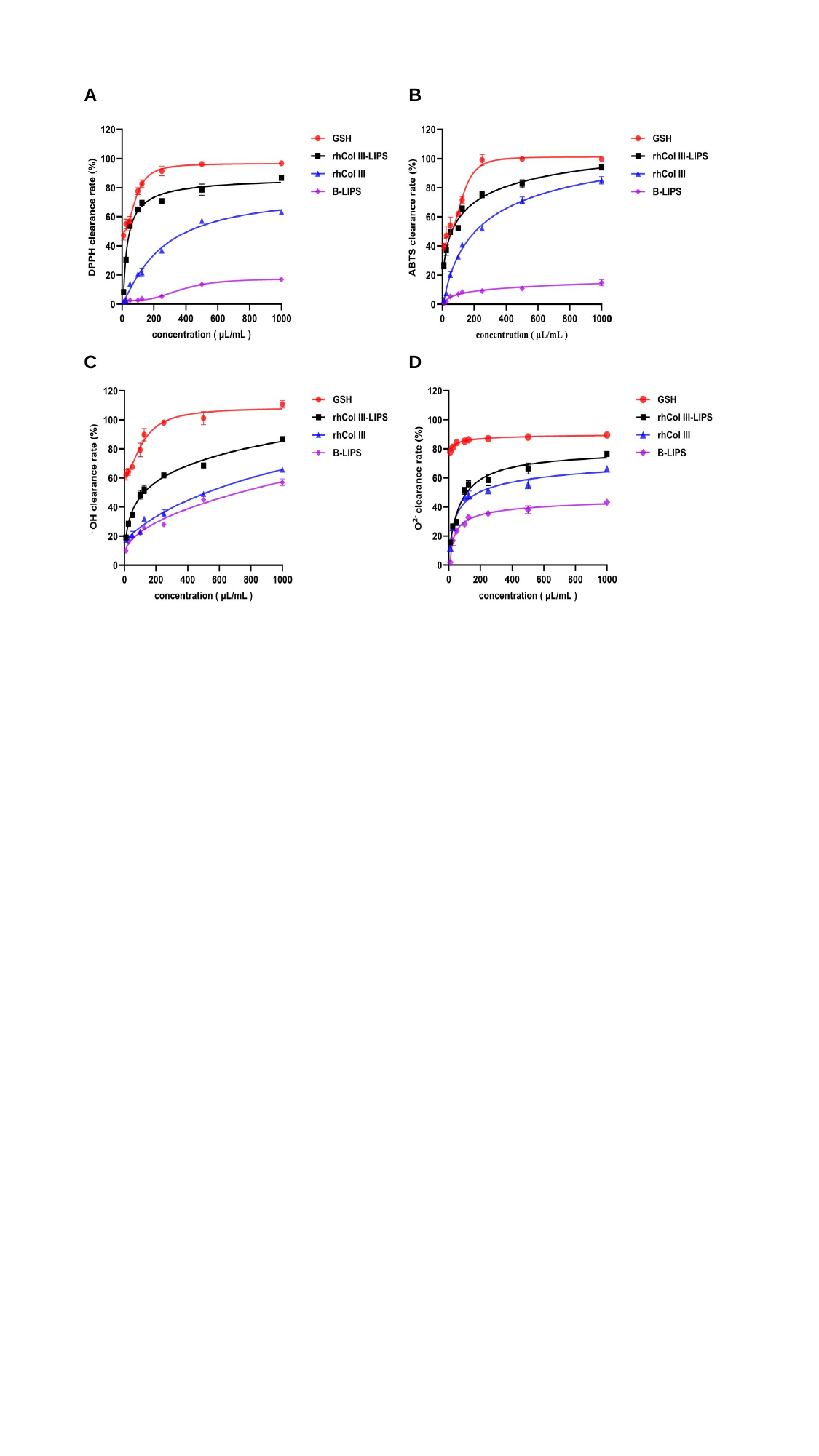

A
B
C
D

## Slide 4
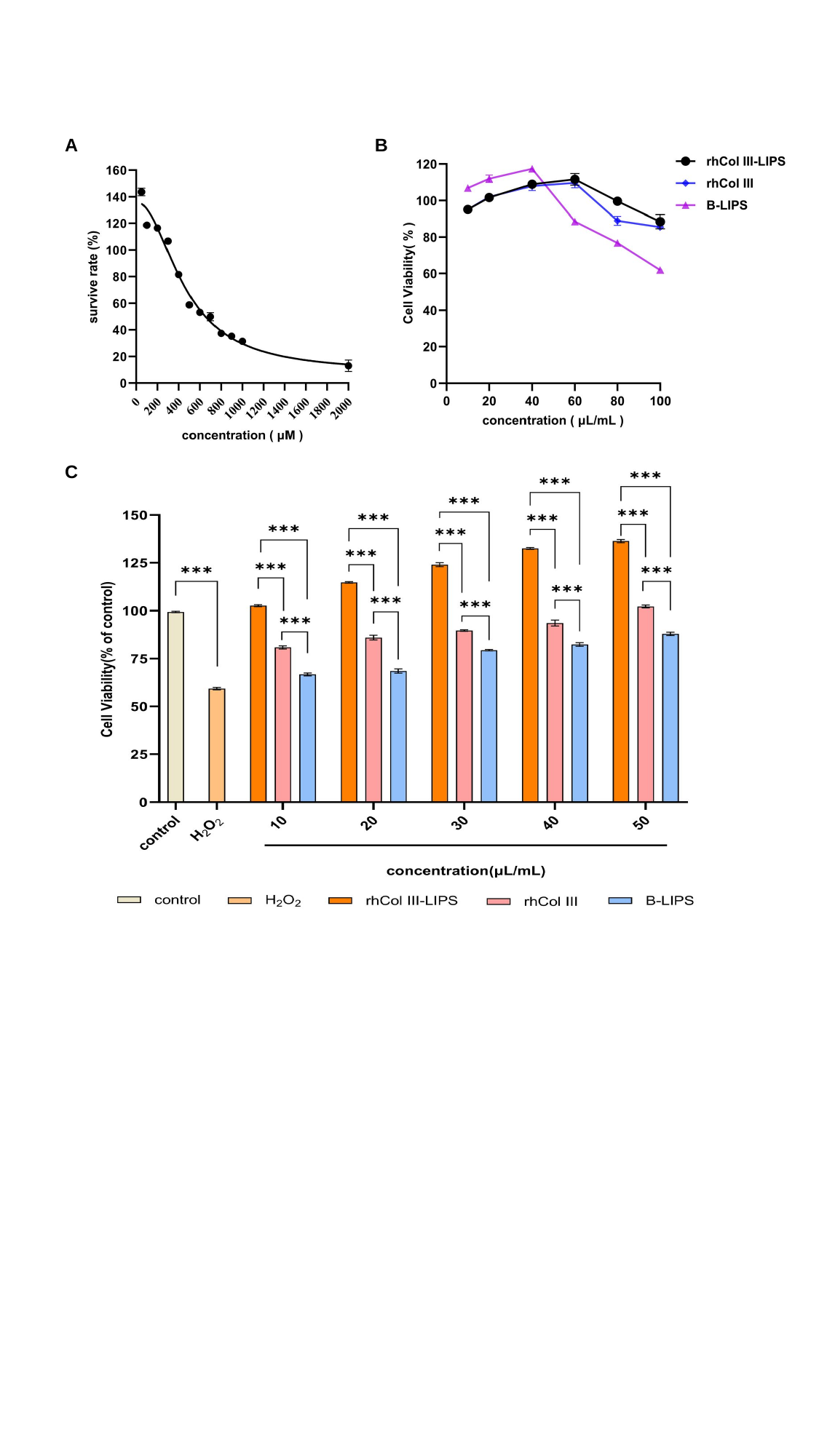

A
B
C

## Slide 5
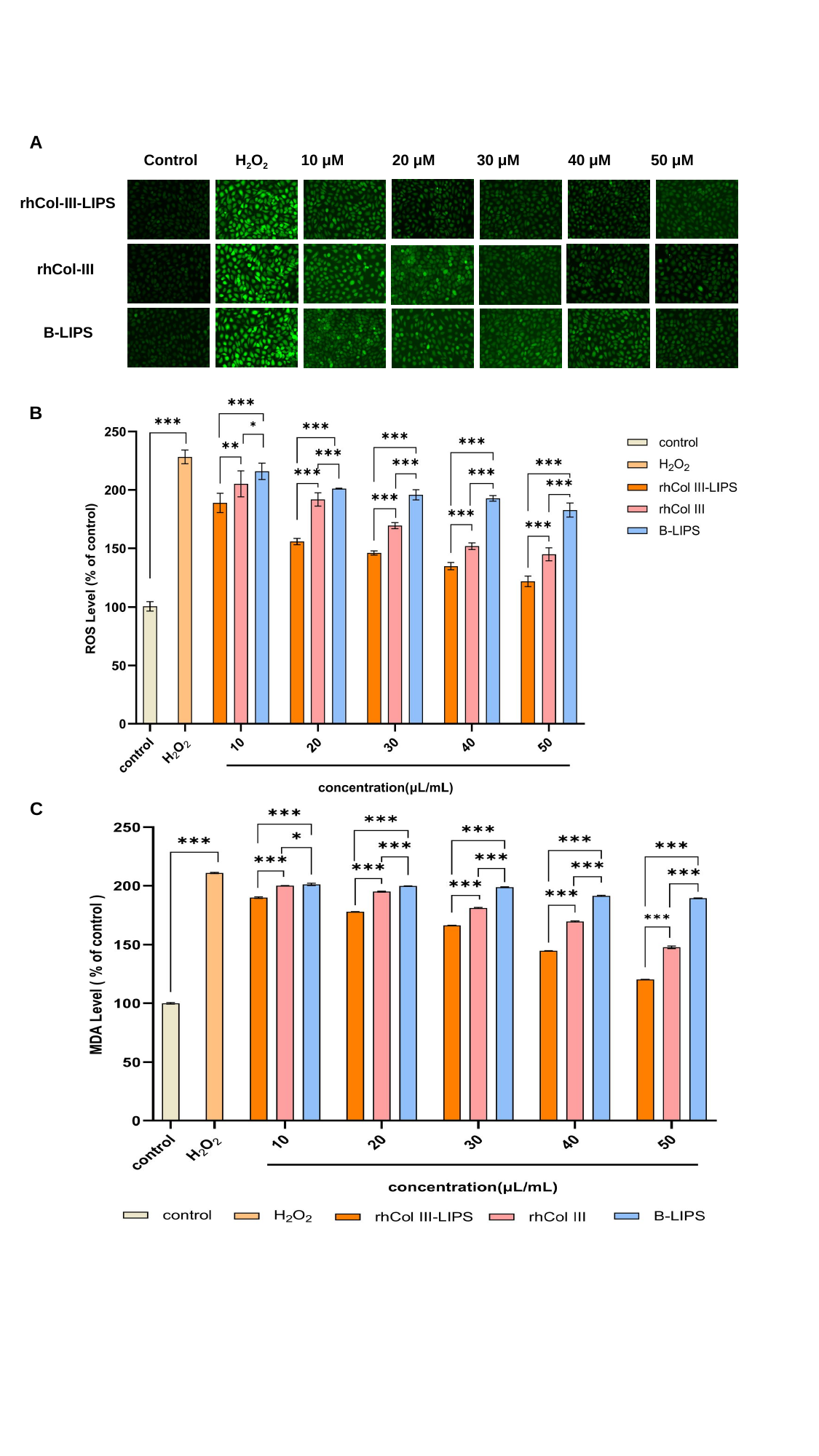

A
Control H2O2 10 μM 20 μM 30 μM 40 μM 50 μM
rhCol-III-LIPS
rhCol-III
B-LIPS
B
C

## Slide 6
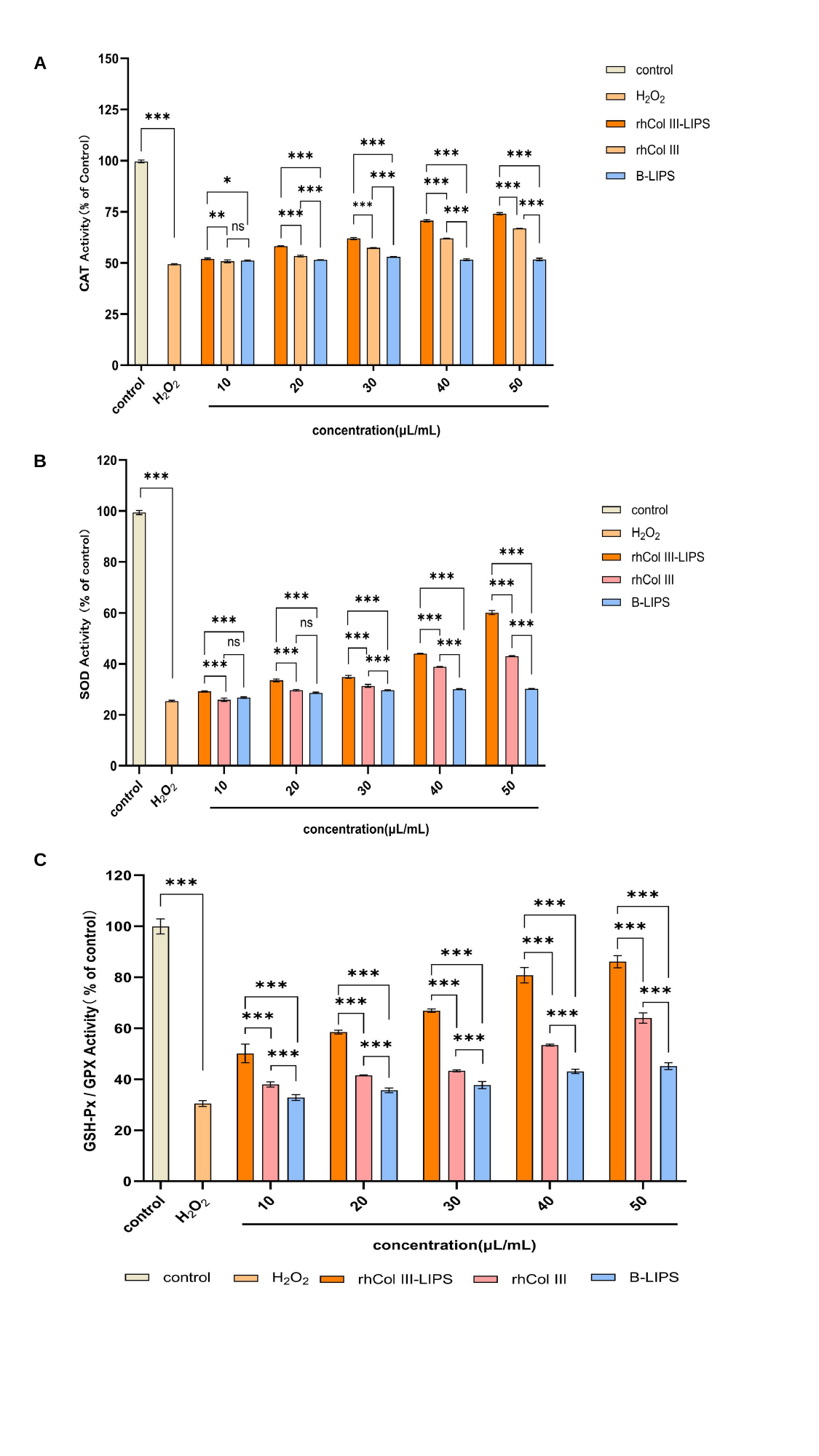

A
B
C
